# Data-driven multiple-level analysis of gut-microbiome-immune-joint interactions in rheumatoid arthritis

**DOI:** 10.1186/s12864-019-5510-y

**Published:** 2019-02-11

**Authors:** QuanQiu Wang, Rong Xu

**Affiliations:** Department of Population and Quantitative Health Science, School of Medicine, Case Western Reserve University, Cleveland, OH 44106 USA

**Keywords:** Gut microbiota, Metabolism, Rheumatoid arthritis, Computational analysis

## Abstract

**Background:**

Rheumatoid arthritis (RA) is the most common autoimmune disease and affects about 1% of the population. The cause of RA remains largely unknown and could result from a complex interaction between genes and environment factors. Recent studies suggested that gut microbiota and their collective metabolic outputs exert profound effects on the host immune system and are implicated in RA. However, which and how gut microbial metabolites interact with host genetics in contributing to RA pathogenesis remains unknown. In this study, we present a data-driven study to understand how gut microbial metabolites contribute to RA at the genetic, functional and phenotypic levels.

**Results:**

We used publicly available disease genetics, chemical genetics, human metabolome, genetic signaling pathways, mouse genome-wide mutation phenotypes, and mouse phenotype ontology data. We identified RA-associated microbial metabolites and prioritized them based on their genetic, functional and phenotypic relevance to RA. We evaluated the prioritization methods using short-chain fatty acids (SCFAs), which were previously shown to be involved in RA etiology. We validate the algorithms by showing that SCFAs are highly associated with RA at genetic, functional and phenotypic levels: SCFAs ranked at top 3.52% based on shared genes with RA, top 5.69% based on shared genetic pathways, and top 16.94% based on shared phenotypes. Based on the genetic-level analysis, human gut microbial metabolites directly interact with many RA-associated genes (as many as 18.1% of all 166 RA genes). Based on the functional-level analysis, human gut microbial metabolites participate in many RA-associated genetic pathways (as many as 71.4% of 311 genetic pathways significantly enriched for RA), including immune system pathways. Based on the phenotypic-level analysis, gut microbial metabolites affect many RA-related phenotypes (as many as 51.3% of 978 phenotypes significantly enriched for RA), including many immune system phenotypes.

**Conclusions:**

Our study demonstrates strong gut-microbiome-immune-joint interactions in RA, which converged on both genetic, functional and phenotypic levels.

## Background

Rheumatoid arthritis (RA) is one of the most common autoimmune diseases and affects over 1.3 million Americans and 1% of the worldwide population ((https://www.rheumatoidarthritis.org/ra/facts-and-statistics/), [1]). RA is complex, with genetic, epigenetic, and environmental factors contributing to disease susceptibility and progression [2]. While significant progress has been made in understanding genetic, molecular, and cellular aspects of RA, relatively little is known about which environmental factors are important in RA susceptibility and how they interact with host genetics in the development of RA [3]. Human and mouse model microbiome studies have shown that gut dysbiosis, an imbalance in the intestinal microbiota [4], is associated with RA [5–11]. Studies in mouse models have shown a requirement of gut microbiota for arthritis development [12–14].

Human gut microbiota contribute to human diseases and health via the cumulative effects of microbial metabolites [15–17]. It has become increasingly clear that the prodigious metabolite activities of gut microbiota strongly influence RA susceptibility and progression [6, 7, 18–21]. Short chain fatty acids (SCFA) are the primary end-products of fermentation of non-digestible dietary fiber by the gut microbiota. SCFAs have emerged as major mediators in linking nutrition, gut microbiota, and human health [22, 23]. Recent studies have shown that SCFAs play important roles in the suppression of inflammation in RA [19]. Mice deficient for SFCA receptor showed exacerbated inflammation in modes of RA [19]. Butyrate, one of the most abundant SCFAs, acts as an endogenous histone deacetylase (HDAC) inhibitor and has been shown to decrease inflammation in animal models of RA and other inflammatory diseases [20].

Although the link between microbial metabolism and RA has been recognized, the mechanisms underlying how microbial metabolites interact with human genetics in promoting or protecting against RA remain largely unknown. We previously demonstrated that data-driven computational approaches have potential in uncovering mechanistic links between microbial metabolites and human diseases (colorectal cancer and Alzheimer’s disease) [24–26]. Specific for RA, we previously developed a mechanism-based prediction system, mMetabolitePredict, for human metabolome biomarker discovery and applied it to identify and prioritize metabolomic biomarkers for RA [27]. We found that among 259,170 prioritized chemicals/metabolites in human body, the subset of metabolites originated from human gut microbiota ranked highly [27]. This finding motivated our current study (funded by Pfizer ASPIRE Rheumatology and Dermatology Research Award) to perform data-driven systematic analysis of which and how human gut microbial metabolites are involved in the immune-joint axis of the RA etiology at the genetic, functional and phenotypic levels. We evaluated the algorithms using SCFAs, which are known to have a role in the suppression of inflammation in RA [19, 20]. We evaluated whether SCFAs were ranked highly based on their genetic, functional and phenotypic relevance to RA. To the best of our knowledge, our study represents the first computational approach to comprehensively characterize the complex gut-microbiome-immune-joint interactions in RA. The unique informatics contribution is that we innovatively leveraged large amounts of publicly available data collected for other purpose and developed data-driven computational methods to understand gut-microbiome-gene-disease interactions. Our approaches are highly flexible and can be applied to any other diseases. The biomedical contribution of our study is that the identification of gut microbial metabolites and the understanding of their role in RA has potential in providing new insights into the basic mechanisms of disease etiology and enable new possibilities for disease diagnosis, prevention, and treatment.

## Results

### Genetic connections: Microbial metabolites may be genetically involved in RA and interact with many RA-associated genes

For evaluation, we show that the genetics-based ranking algorithm ranked all three SCFAs consistently highly across three complementary disease genetics data resources (Table 1). SCFAs on average ranked in the top 3.52% among 127 gut microbial metabolites, with acetic acid ranked at top 1, butyric acid at top 2 and propionic acid at top 9. Our study shows that butyric acid regulates many RA-related genes, including IL10, IL2, IL6, and STAT4 (Table 2), suggesting the potential roles of SCFAs for their anti-inflammatory effects in protecting joint in RA.

**Table 1:** Evaluation of genetic associations between RA and SCFAs (butyric acid, acetic acid, and propionic acid). RA-associated genes from three disease genetics resources (OMIM, the GWAS Catalog, and ClinVar) were used separately and combined

Disease Genetics	Recall	Mean Ranking (top %)	Median Ranking (top %)	*P*-value
OMIM (15 RA genes)	1.00	4.61	4.07	9.96E-4
GWAS (155 RA genes)	1.00	4.33	2.44	0.0036
ClinVar (10 RA genes)	1.00	4.61	4.07	9.96E-4
Combined (166 RA genes)	1.00	3.52	2.44	0.0017

**Table 2:** Top ten microbial metabolites ranked based on shared genes with RA (166 genes from combined resources were used). SCFAs are highlighted

Metabolite	Targeted RA Genes	Targeted RA Genes
Acetic acid	30	ACP5, ANKRD55, BAG6, BLK, CDK6, CIITA, CLYBL, CSF2 GABARAPL3, GATA3, GRM5, HLA-DQA1, HLA-DQB1 IL10, IL2, IL2RB, IL6, KCNIP4, MECP2, NFKBIE, NOTCH4 PPIL4, RAD51B, REL, SUOX, TEC, TXNDC11, TYK2 UTS2, ZNF774
Butyric acid	13	ACOXL, CDK6, CLYBL, CSF2, GRM5, IL10, IL2, IL6 MECP2, PRKCB, PRKCH, STAT4, UTS2
Acetaldehyde	13	CSF2, DPP4, HLA-DRB1, IL6, KCNIP4, PADI4, PPIL4 PRKCB, RAD51B, STAG1, TRNT1, TXNDC11, ZNF774
Methane	12	BAG6, CSF2, CTLA4, EOMES, GATA3, GRM5, IFNAR1 IL10, IL2, IL6, PTPN22, STAG1
mannitol	10	ARHGEF3, BAG6, BLK, CLYBL, IL6, LRRC18, SLC22A4 TEC, TNFAIP3, TYK2
1-butanol	8	ANKRD55, BAG6, IL2, IL6, NFKBIE, NOTCH4 PTPN2, PTPN22
Isopropyl alcohol	8	ACP5, CLYBL, GCH1, IL10, IL6, KCNIP4 PTPN2, PTPN22
Propionic acid	7	GATA3, GCH1, GRM5, IL10, IL6, SLC22A4, UTS2
Succinic acid	6	BAG6, CCL21, CCR6, CXCR5, OS9, PPIL4
Isobutyric acid	6	CLYBL, GCH1, NFKBIE, REL

Our studies show that 61 out of the 127 gut microbial metabolites directly interact with RA-associated genes. The top 10 microbial metabolites (ranked based on the number of shared genes with RA) and their shared RA gene are shown in Table 2. For example, acetic acid ranked at top 1 and shared 30 genes with RA (18.1% of all 166 RA genes). Many of the shared genes are immune-related, strongly suggesting the gut-microbiome-immune-joint interactions in RA.

### Functional connections: Microbial metabolites may be functionally involved in RA and participate in many RA-associated genetic pathways

We identified genetic pathways significantly associated with RA and for each microbial metabolite. We then ranked metabolites based on the numbers of shared genetic pathways with RA. All three SCFAs ranked highly based on their pathway overlaps with RA (Table 3). A total of 311 pathways were significantly enriched for RA, among which butyric acid shared 222 pathways (71.4%), acetic acid shared 126 pathways (40.5%), and propionic acid shared 152 pathways (48.9%). Among 127 microbial metabolites, 116 metabolites shared at least one genetic pathway with RA. The top 20 ranked metabolites are shown in Table 4. The fact that the majority of RA-associated genetic pathways were regulated by SCFAs and other gut microbial metabolites indicates that human gut microbial metabolism is functionally involved in RA etiology.

**Table 3:** Evaluation of functional associations between RA and SCFAs (butyric acid, acetic acid, and propionic acid). RA-associated genes from three disease genetics resources (OMIM, the GWAS Catalog, and ClinVar) were used separately and combined

Disease Genetics	Recall	Mean Ranking (top %)	Median Ranking (top %)	*P*-value
OMIM (15 RA genes)	1.00	5.96	5.69	0.0023
GWAS (155 RA genes)	1.00	5.69	5.69	0.0018
ClinVar (10 RA genes)	1.00	5.69	5.69	0.0018
Combined (166 RA genes)	1.00	5.69	5.69	0.0018

**Table 4:** Top 20 microbial metabolites ranked based on shared genetic pathways. SCFAs are highlighted

Rank	Metabolite	Shared Pathways (n)	Rank	Metabolite	Shared Pathways (n)
1	Methane	232	11	**Acetic acid**	126
2	Mannitol	227	12	Trehalose 6-phosphate	125
3	**Butyric acid**	222	13	5-aminopentanoic acid	118
4	Benzoyl-coa	169	14	Isobutanol	118
5	Trehalose	163	15	Hydroxyphenyllactic acid	115
6	1-butanol	153	16	Piperidine	107
7	**Propionic acid**	152	17	Phenylacetic acid	107
8	Isopropyl alcohol	148	18	Acetaldehyde	98
9	Trans-ferulic acid	139	19	2,3-butanediol	98
10	Chenodeoxycholic acid glycine conjugate	136	20	Acetone	90

We then ranked the shared pathways between RA and each metabolite by the balanced measure of their enrichment folds. For example, the pathway “*IL27-mediated signaling events*” was 10.31-fold enriched for RA and 6.48-fold enriched for butyric acid. The F1 combined enrichment fold of this pathways was 7.96. The top 10 genetic pathways regulated by both RA and butyric acid are shown in Table 5. As shown in the table, the majority of the top shared pathways are related to immune functions, strongly suggesting the gut-microbiome-immune interactions in RA at functional-level. We performed the same analysis for the other two SCFAs (acetic acid and propionic acid) and for methane (the top one ranked and non-SCFA metabolite). Table 6 shows the top ten ranked pathways shared between RA and acetic acid, propionic acid and methane. The results show that majority of the top shared pathways between RA and metabolites (SCFAs and non-SCFA methane) are related to immune functions, though the specific pathways for each metabolite vary. In summary, microbial metabolites may be involved in RA pathology through different immune pathways.

**Table 5:** Top 10 shared genetic pathways shared between RA and butyric acid

Pathway	Enrichment Fold (RA)	Enrichment fold (butyric acid)	Combined
IL27-mediated signaling events	10.31	6.48	7.96
IL-10 Anti-inflammatory Signaling Pathway	9.47	6.60	7.78
Th1/Th2 Differentiation	16.94	4.73	7.39
Cytokines and Inflammatory Response	9.25	5.03	6.52
NO2-dependent IL 12 Pathway in NK cells	9.47	4.62	6.21
Erythrocyte Differentiation Pathway	7.15	5.24	6.05
IL 2 signaling pathway	7.31	5.10	6.01
Cytokine Network	7.31	5.10	6.01
IL22 Soluble Receptor Signaling Pathway	6.70	4.91	5.67
Regulation of hematopoiesis by cytokines	10.73	3.74	5.55

**Table 6:** Top 10 shared genetic pathways between RA and other SCFAs (acetic acid, propionic acid) and methane (top one ranked metabolite)

RA ∩ Acetic acid	RA ∩ Propionic acid	RA ∩ Methane
IL 2 signaling pathway	Interleukin-6 signaling	Regulation of hematopoiesis by cytokines
Trka Receptor Signaling Pathway	IL 5 Signaling Pathway	Cytokine Network
IL 5 Signaling Pathway	Antigen Dependent B Cell Activation	Dendritic cells in regulating TH1 and TH2 Development
Cadmium induces DNA synthesis and proliferation in macrophages	Organic cation/anion/zwitterion transport	Antigen Dependent B Cell Activation
Role of ERBB2 in Signal Transduction and Oncology	Dendritic cells in regulating TH1 and TH2 Development	Cytokines and Inflammatory Response
Interleukin-6 signaling	Trafficking of GluR2-containing AMPA receptors	IL 17 Signaling Pathway
IL2 signaling events mediated by STAT5	Regulation of hematopoiesis by cytokines	IL 2 signaling pathway
RB Tumor Suppressor/Checkpoint Signaling in response to DNA damage	Role of ERBB2 in Signal Transduction and Oncology	STAT3 Pathway
IL2 signaling events mediated by PI3K	Folate biosynthesis	Activation of Csk by cAMP-dependent Protein Kinase Inhibits Signaling through the T Cell Receptor
Interleukin 13 (IL-13) Pathway	Cytokine Network	GATA3 participate in activating the Th2 cytokine genes expression

### Phenotypic connections: Microbial metabolites may affect RA at the phenotypic level and affect many RA-related phenotypes

We examined the phenotypic connections between gut microbial metabolites and RA. As the evaluation, we showed that SCFAs were significantly associated with RA at the phenotypic level (Table 7). The top 20 metabolites ranked based on phenotypic overlaps with RA are shown in Table 8. For example, a total of 978 phenotypes were significantly enriched for RA-associated genes (166 genes from combined resources), among which butyric acid shared 502 phenotypes (51.3%) and propionic acid shared 335 phenotypes (34.3%) with RA. These results indicate that SCFAs and other microbial metabolites are phenotypically involved in RA.

**Table 7:** Evaluation of phenotypic associations between RA and SCFAs. RA-associated genes from three disease genetics resources (OMIM, the GWAS Catalog, and ClinVar) were used separately and combined

Disease Genetics	Recall	Mean Ranking (top %)	Median Ranking (top %)	*P*-value
OMIM (15 RA genes)	1.00	15.00	5.00	0.1022
GWAS (155 RA genes)	1.00	23.33	7.50	0.2923
ClinVar (10 RA genes)	1.00	14.72	5.00	0.0969
Combined (166 RA genes)	1.00	16.94	5.83	0.1300

**Table 8:** Top 20 ranked microbial metabolites based on shared phenotypes. SCFAs are highlighted

Rank	Metabolite	Shared Phenotypes (n)	Rank	Metabolite	Shared Phenotypes (n)
1	methane	533	11	5-aminopentanoic acid	296
2	**butyric acid**	502	12	trans-ferulic acid	241
3	isopropyl alcohol	386	13	piperidine	240
4	**mannitol**	371	14	indoxyl sulfate	230
5	benzoyl-coa	340	15	phenylethylamine	211
6	1-butanol	337	16	putrescine	208
7	**propionic acid**	335	17	zeaxanthin	196
8	trehalose	314	18	muramic acid	193
9	isobutanol	313	19	succinic acid	189
10	2-hydroxyglutarate	304	20	chenodeoxycholic acid glycine conjugate	184

### Case study: Butyric acid is phenotypically involved in RA

Butyric acid is the most abundant metabolites of gut microbiota in the fermentation of dietary fiber. Our above analysis showed that butyric acid was highly associated with RA at both genetic, functional and phenotypic levels. We then examined how butyric acid was phenotypically involved in RA. Butyric acid shared 502 phenotypes with RA. We classified these shared phenotypes based on the Mouse Phenotype Ontology (MPO) classification schemes (3rd- and 4th-level classifications). These 502 phenotypes were classified into 52 3rd-level classes and 164 4th-level classes.

**Fig. 1:**
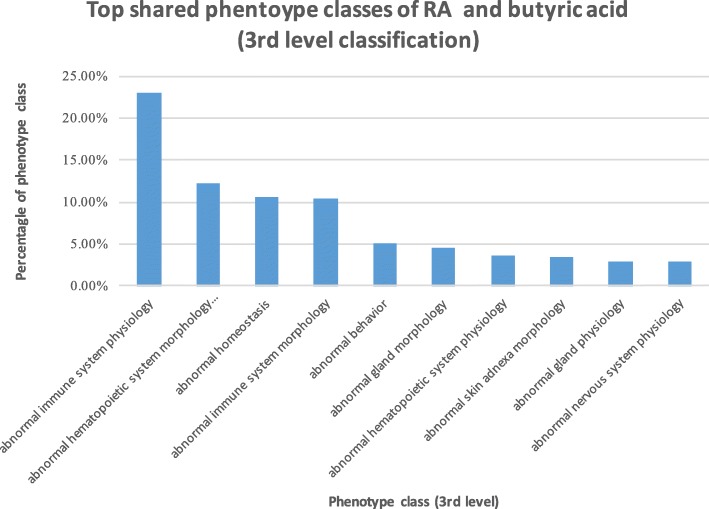
Top 10 ranked 3rd-level classes of phenotypes shared between RA and butyric acid

**Fig. 2:**
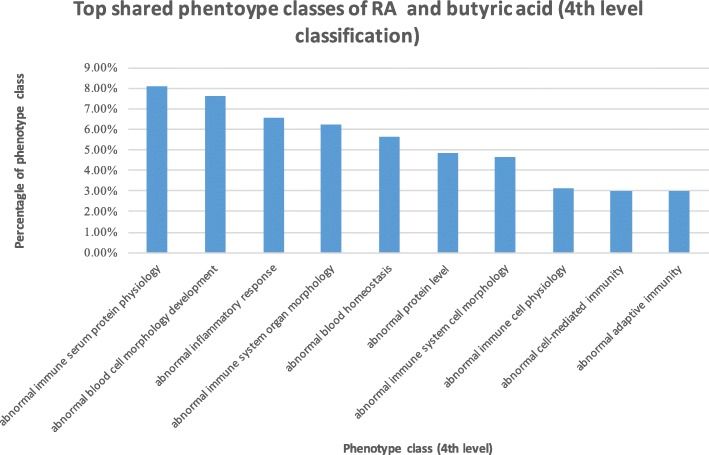
Top 10 ranked 4th-level classes of phenotypes shared between RA and butyric acid

The top 10 3rd-level classes are shown in Fig. 1. The 3rd-level phenotype class “*abnormal immune system physiology*” ranked at top one. Among a total of 502 phenotypes shared between RA and butyric acid, 148 (23%) phenotypes belonged to this class. The top ten 4th-level classes of the shared phenotypes are shown in Fig. 2, among which seven phenotypes were directly related to immune functions, including “*abnormal immune serum protein physiology*”, “*abnormal inflammatory response*”, “*abnormal cell-mediated immunity*” and “*abnormal adaptive immunity*”.

Since the gene-phenotype annotations in MGI are largely mutational, our phenotypic-level analysis suggests potential causal phenotypic effects of butyric acid and other gut microbial metabolites on RA, though effects on various immune functions.

## Discussions

In this study, we performed data-driven analysis of the gut-microbiome-immune-joint interactions in RA. We showed that gut microbial metabolites were strongly involved in RA at both genetic, functional and phenotypic levels. Specifically, our study shows that gut microbial metabolites interact with many RA-associated genes, participate in RA-related immune pathways and affect RA-associated immunological phenotypes. As compared to our previous studies for colorectal cancer and Alzheimer’s diseases, the identified microbial metabolites as well as the genetic pathways involved are different in RA from ones in colorectal cancer and Alzheimer’s disease [24–26]. For example, SCFAs are highly related to RA through immune pathways. On the other hand, trimethylamine-n-oxide is highly related to colorectal cancer through many cancer-related pathways [24, 26]. To the best of our knowledge, our study represents the first computational approach to comprehensively characterize the complex gut-microbiome-immune- joint interactions in RA. While our study is pure ‘in silico’, it generated large amounts of data/knowledge/hypotheses that can facilitate other biomedical researchers to conduct hypothesis-driven functional studies of gut microbial metabolisms in RA. A few limitations inherent in the publicly available datasets warrant further discussion.

First, although our analysis suggests strong functional connections between gut microbial metabolites and RA, especially in our phenotypic analysis that used the causal/mutational gene-phenotype associations, our findings are largely associational. In order to translate the findings for RA diagnosis, prevention, and treatment, it is necessary to establish cause-effect relationships of the identified metabolite-gene-pathway-phenotype-RA associations and identify specific gut bacteria that produce the metabolites.

Second, HMDB is currently the most comprehensive human metabolome database, containing a total of 83,479 small molecule metabolites found in the human body. STITCH is currently the most comprehensive chemical genetics databases, containing genetic associations for 500,000 small molecules. However, HMDB contains only 172 metabolites originated from gut microbiota, among which 127 metabolites have associated genes in STITCH. The field of human microbiome research is fast moving, with an increasing number of microbial metabolites being identified and published in literature. We are currently developing text mining and natural language processing techniques to extract human gut microbial metabolites from published biomedical literature.

Third, our data-driven multi-level analysis is not specific to RA and can be applied to any other diseases. The only change to the algorithm is to replace RA-associated genes to genes associated with another disease. However, the lack of evaluation data (known microbial metabolites associated with diseases) in structured format prevented us from systematically evaluating how the algorithm perform in other diseases. Increasing number of published biomedical research articles have reported the associations among microbial metabolites, gut bacteria, and diseases. However, the knowledge is still buried in free-text documents with limited machine understandability. In order to systematic evaluate our algorithm in other diseases, we need either manually curate or develop natural language processing techniques to automatically extract disease-microbial metabolite associations from biomedical literature. We are actively pursuing the latter approach.

Fourth, studies have shown the importance of diet and associated changes in the gut microbiota in human diseases. Intestinal SFCAs are produced by gut microbiota digesting high fiber diet and are involved in human metabolism and health [22, 23]. Trimethylamine n-oxide (TMAO) is formed by gut microbiota in digesting red meat and high fat diet. Both human studies have shown that TMAO is mechanistically involved in cardiovascular diseases [28], renal disease [29] and colorectal cancer [30]. However, the exact relationship among specific diet, bacteria and diseases remain largely unknown. In one of our recent studies, we developed network-based systems approach to investigate food-metabolite interactions in Alzheimer’s disease [31]. The food metabolites can be produced by either human host or gut bacteria, however we currently lack the knowledge to differentiate these two. The identification and understanding how diet and food are associated with diseases by impacting gut microbiota will have great potential in treating and preventing human diseases, including RA.

The computational framework that we developed has built-in flexibility and capability for us to continuously incorporate new data as it becomes available in our future studies. We believe that our view of gut-microbiome-RA interactions will evolve as more data becomes available.

## Conclusions

The cause of RA remains largely unknown and could result from a complex interaction between genes and environment factors. Recent studies suggested that gut microbiota and their collective metabolic outputs exert profound effects on the host immune system and are implicated in RA. However, which and how gut microbial metabolites interact with host genetics in contributing to RA pathogenesis remains unknown. In this study, we present a data-driven study to understand how gut microbial metabolites contribute to RA at the genetic, functional and phenotypic levels. Our in-silico data-driven study suggests strong gut-microbiome-immune-joint interactions in RA, which converge on both genetic, functional and phenotypic levels.

## Methods

We used publicly available human disease genetics, human chemical genetics, human metabolome, genetic signaling pathways, mouse genome-wide mutation phenotypes, and mouse phenotype ontology to characterize the genetic, functional, and phenotypic connections between human gut microbial metabolites and RA.

### RA genetics data

We used three data resources to obtain RA-associated genes: (1) we obtained 155 RA-associated genes form the Catalog of Published Genome-Wide Association Studies (GWAS catalog) (data accessed in June, 2017). The GWAS catalog is an exhaustive source of disease/trait-gene associations from published GWAS data and currently contains 34,790 disease/trait-gene pairs for 1655 common complex diseases/traits [32], 2) we obtained 16 RA-associated genes from the Online Mendelian Inheritance in Man database (OMIM) (data accessed in July, 2017). OMIM is the most comprehensive source of disease genetics for Mendelian disorders and currently includes 10,125 disease-gene pairs for 10,674 diseases/phenotypes [33]; and (3) we obtained 10 RA-associated genes from ClinVar (data accessed in July, 2017). ClinVar is a publicly available resource of reports of the relationships among human variations and phenotypes and currently contains 9873 disease-gene associations for 5240 diseases/phenotypes [34]. We used these three complementary disease genetics resources to demonstrate the robustness of our findings.

### The human metabolome database (HMDB)

HMDB contains detailed information about small molecule metabolites found in the human body [35]. Currently, HMDB contains 83,479 metabolites. In this study, we focused on the 172 metabolites originated in human gut microbiota (data accessed in July, 2017).

### Metabolite genetics data

We used STITCH (Search Tool for Interactions of Chemicals) database to obtain genes associated with gut microbial metabolites obtained from HMDB. STITCH contains data on the interactions between 500,000 small molecules and 9.6 million proteins from 2031 organisms [36]. In this study, we used chemical-gene associations found in human body, which include 15,473,939 chemical-gene pairs for 473,602 chemicals, and 18,701 human genes (data accessed in July, 2017). Among the 172 microbial metabolites from HMDB, 127 were mapped.

to chemical names in STITCH. Genes associated with the mapped microbial metabolites were then obtained from STITCH. For example, we mapped butyric acid (in HMDB) to butyrate (in STITCH) and obtained a total of 793 butyrate-associated genes from STITCH.

### Molecular pathway data

We used rich pathway information from the Molecular Signatures Database (MSigDB) to investigate how microbial metabolites were functionally related to RA. MSigDB is currently the most comprehensive resource of 17,779 annotated pathways and gene sets [37] (data accessed in July, 2017). For each microbial metabolite, we identified molecular pathways that were significantly enriched for both RA and the metabolite.

### Genome-wide mutational phenotypes in mouse models and mouse phenotype ontology

The Mouse Genome Database (MGD) made available large amounts of phenotypic descriptions of systematic genetic knockouts in mouse models [38]. Such large-scale systematic genetic knockouts are impossible to do in human. These strong causal gene-phenotype associations (318,709 gene-phenotype association annotations for 60,474 targeted mutant alleles and 12,104.

phenotypes) have been useful for screening functional effects of chemicals on disease phenotypes. We have recently developed computational algorithms to perform virtual phenotypic screening to prioritize drugs for diseases by matching mouse mutational phenotype profiles between drugs and diseases [39–42]. We validated our virtual screening drug candidates in experimental models of ovarian cancer [39, 40]. In this study, we used the same strategy to assess the phenotypic effects of gut microbial metabolites on RA-related phenotypes. For example, the microbial metabolite butyrate is associated with the gene IL17A and the knockout of IL17A in mouse models is associated with the phenotype “*rheumatoid arthritis*”. We then used the classification scheme of the Mouse Phenotype Ontology (MPO) at MGD to group identified phenotypes (e.g., “*rheumatoid arthritis*”, “*abnormal cytokine level*”) into classes (e.g., “*abnormal immune system physiology*”).

### Analyze genetic connections between gut microbial metabolites and RA and prioritize metabolites based on their shared genes with RA

We hypothesize that if a metabolite interacts with many RA-associated genes, then this metabolite may be highly involved in RA. We prioritized microbial metabolites based on number of their shared genes with RA. We obtained RA-associated genes from three complementary disease genetics data resources (OMIM, the GWAS Catalog, and Clinvar): 15 RA-associated genes from OMIM, 155 genes from the GWAS Catalog, 10 genes from ClinVar, and 166 genes from these three resources combined. We obtained microbial metabolite-associated genes from the STITCH database. Metabolites were ranked based on the numbers of their shared genes with RA.

### Evaluation

Animal studies showed that SCFAs had a role in the suppression of inflammation in RA [19, 20]. For evaluation, we examined whether SCFAs were ranked highly based on their genetic overlaps with RA. Both mean and median rankings of SCFAs among all metabolites were calculated. Significance was calculated by comparing mean rankings to random expectation, which is 50%. Rankings based on three disease genetics data resources were compared to demonstrate the robustness of our analysis and findings.

### Analyze functional connections between gut microbial metabolites and RA and prioritize metabolites based on their shared genetic pathways with RA

We obtained RA-associated genes from the three disease genetics databases separately. Pathways associated with each gene were obtained from MSigDB. For each pathway, we assessed its probability of being associated with a given set of RA-associated genes (e.g., 15 RA genes from the OMIM database) as compared to its probability of being associated with the same number of randomly selected genes. The random process was repeated 1000 times and a t-test was used to assess the statistical significance. For instance, a total of 108 pathways were significantly associated with the 16 RA-associated genes from OMIM. The pathway “*Cytokines and Inflammatory Response*” had a significant 61-fold enrichment as compared to the random expectation. Similarly, we identified significantly enriched genetic pathways for each of the 127 microbial metabolites (with metabolite-associated genes obtained from the STITCH database). For example, a total of 748 pathways were significantly enriched for butyric acid. The pathway “*Cytokines and Inflammatory Response*” shows a significant 5-fold enrichment for butyric acid.

A shared genetic pathway may be associated with RA and a metabolite at different significance level. We developed a prioritization measure to identify shared pathways that rank highly for both RA and a metabolite. The intuition is that a shared pathway between RA and a metabolite ranks highly if and only if it ranks highly for both RA and the metabolite. The ranking of a shared pathway between RA and a metabolite s defined as: *rank* = 2*(ranking_ra * ranking_m)/(*ranking_ra* + 2 *ranking_m*), where *ranking_ra* is the enrichment fold of a pathway for RA; and *ranking_m* is the enrichment fold of the same pathway for the metabolite. For example, the pathway “cytokines and inflammatory response” showed a 61-fold enrichment for RA and a 5-fold enrichment for butyric acid. The combined ranking score of this shared pathway for both RA and butyric acid was 9.24. After identifying shared pathways, metabolites were then prioritized based on the numbers of shared pathways with RA.

### Evaluation

The prioritization algorithm was evaluated using three known RA-associated SCFAs. Mean ranking, median rankings and the significance were calculated. Rankings based on three different disease genetics data resources were compared to demonstrate the robustness of the finding.

### Analyze phenotypic connections between gut microbial metabolites and RA and prioritize metabolites based on their shared phenotypes with RA

We obtained RA-associated genes to their corresponding mouse gene homologs (e.g., *IL17A = > Ctla)* using human-mouse homolog mapping data from MGD [34]. The mapped mouse genes were then linked to their corresponding mutational phenotypes in mouse models (e.g., *IL17A = > rheumatoid arthritis*, *TNF = > abnormal inflammatory response*) using gene-phenotype association annotations from MGD. For each mapped phenotype, we assessed its probability of being associated with RA-associated genes as compared to its probability associated with the same number of randomly selected genes. The random process was repeated 1000 times and a t-test was used to assess the statistical significance. As an example, the phenotype “*abnormal T-helper 1 physiology*” showed a significant 36-fold enrichment for RA as compared to random expectation. Similarly, we identified significantly enriched phenotypes for each gut microbial metabolite. For example, the phenotype “*abnormal T-helper 1 physiology*” shows a significant 1.7-fold enrichment for butyric acid. Phenotypes shared between RA and each metabolite were then prioritized as described above for prioritizing shared genetic pathways. After identifying shared phenotypes, metabolites were then prioritized based on the numbers of shared phenotypes with RA.

### Evaluation

The prioritization algorithm was evaluated using three known RA-associated SCFAs. Mean ranking, median rankings and the significance were calculated. Rankings based on three different disease genetics data resources were compared to demonstrate the robustness of the finding.

## Abbreviations

RA: Rheumatoid arthritis
SCFAs: Short-chain fatty acids
HDAC: Histone deacetylase
GWAS catalog: The Catalog of Published Genome-Wide Association Studies
OMIM: The Online Mendelian Inheritance in Man database
HMDB: The Human Metabolome Database
STITCH: Search Tool for Interactions of Chemicals
MSigDB: The Molecular Signatures Database
MGD: The Mouse Genome Database
